# *N*-Butylphthalide Alleviates Blood–Brain Barrier Impairment in Rats Exposed to Carbon Monoxide

**DOI:** 10.3389/fphar.2016.00394

**Published:** 2016-10-26

**Authors:** Mingjun Bi, Mingwei Zhang, Dadong Guo, Weikang Bi, Bin Liu, Yong Zou, Qin Li

**Affiliations:** ^1^Department of Integration of Chinese and Western Medicine, The Affiliated Yantai Yuhuangding Hospital of Qingdao UniversityYantai, China; ^2^Emergency Center, The Affiliated Yantai Yuhuangding Hospital of Qingdao UniversityYantai, China; ^3^Affiliated Shouguang People’s Hospital of Weifang Medical CollegeWeifang, China; ^4^Eye Institute of Shandong University of Traditional Chinese MedicineJinan, China; ^5^Department of Clinical Medicine, Qingdao University Medical CollegeQingdao, China; ^6^The Second Clinical Medical College, Shandong University of Traditional Chinese MedicineJinan, China

**Keywords:** aquaporin-4, blood–brain barrier, claudin-5, CO poisoning, *N*-butylphthalide, Zonula occludens-1

## Abstract

Carbon monoxide (CO) poisoning is one of the most important health concerns and may result in neuropathologic changes and neurologic sequelae. However, few studies have addressed the correlation between CO poisoning and blood–brain barrier (BBB) impairment. In this study, we investigated the effects of *N*-butylphthalide (NBP) on the expressions of zonula occludens-1 (ZO-1), claudin-5 and aquaporin-4 (AQP-4) proteins in a CO poisoning rat model. The results indicated that the brain water content was obviously increased, and the tight junctions between endothelial cells were disrupted, resulting in significant cerebral edema and BBB dysfunction in a rat model of CO poisoning. Meanwhile, the ultrastructure of endothelial cells and pericytes was seriously damaged, and the expressions of ZO-1 and claudin-5 were decreased at an early stage (<7 days). NBP treatment could efficiently maintain the ultrastructural and functional integrity of BBB, alleviate cerebral edema. Besides, NBP could also markedly increase the levels of both ZO-1 and claudin-5 proteins compared with those in rats exposed to CO (*P* < 0.05), whereas NBP had no apparent regulatory effect on AQP-4 expression. Taken together, this study highlights the importance of ZO-1 and claudin-5 proteins in maintaining BBB ultrastructure and function after CO poisoning. NBP, as a novel treatment approach, may effectively inhibit the down-regulation of ZO-1 and claudin-5 proteins (but not AQP-4), thereby preserving the barrier function and reducing cerebral edema after CO poisoning.

## Introduction

Carbon monoxide (CO) poisoning is one of the most important toxicological causes of morbidity and mortality around the world, and the acute brain damage and the delayed encephalopathy are the most serious complications followed by CO poisoning. However, their pathogenesis is poorly understood (Hampson et al., 2009; Geraldo et al., 2014). Currently, there is no efficient treatment and prevention for the neurologic sequelae after CO exposure. Cerebrovascular protection has become a hot topic in recent years. Some scholars put forward the concept of neurovascular unit (NVU), which maintains the physiological function of the normal neurons and the restoration of the damaged neurons (Spindler and Hsu, 2012; Urban et al., 2012; Maki et al., 2013). As a crucial part of NVU, blood–brain barrier (BBB) dysfunction can directly affect the stability of microenvironment in nervous system, and has been known as an important pathological phenomenon of hypoxic-ischemic conditions. The reason why CO is particularly dangerous is that it can bind to hemoglobin, then produces carboxyhemoglobin (HbCO) which results in the displacement of oxygen, and subsequently reduces the supply of oxygen available to the tissues of the body. Thus, the brain damage followed by CO poisoning has many characteristics in common with hypoxic-ischemic disease in pathogenesis (Busl and Greer, 2010; Ahmad and Sharma, 2012), and the BBB damage inevitably occurs in most cases after CO exposure. However, the underlying mechanism has not yet been elucidated.

**FIGURE 1:**
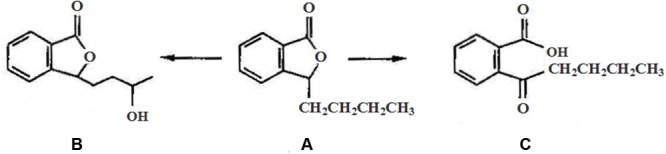
**The chemical structure of NBP and its main metabolites *in vivo*: **(A)***N*-Butylphthalide, **(B)** hydroxylated metabolite of side-chain of NBP, and **(C)** oxidized metabolite of NBP after open loop of lactonic ring**.

Our previous study indicated that the significantly ultrastructural alterations occurred in brain parenchyma in rats after CO exposure, including brain edema, chromatin condensation, nucleus membrane and cell organelles decomposition, even neuronal death (Li Q. et al., 2015; Li et al., 2016). *N*-butylphthalide (NBP), a new drug extracted from celery seeds (see **Figure 1**), was approved by China Food and Drug Administration in 2002. To date, many experiments confirm that NBP treatment could significantly reduce oxidative damage, regulate energy metabolism, and alleviate cerebral edema in acute focal cerebral ischemia rats, protecting mitochondria function from brain damage directly through BBB in patients with acute and chronic cerebral ischemia and dementia (Yang et al., 2015). Meanwhile, NBP had also positive effects in stabilizing the NVUs and improving the neurological deficits through its primary target vascular endothelium (Li et al., 2007; Thored et al., 2007). Besides, the application of NBP could inhibit neuronal apoptosis, and attenuate brain parenchymal injury induced by CO poisoning (Li et al., 2016). Nevertheless, the effects of NBP on the ultrastructural integrity and on the BBB after CO poisoning are still unknown. We infer that NBP treatment could also attenuate the structural and functional abnormality of BBB after CO poisoning. Zonula occludens-1 (ZO-1) and claudin-5 are the most important molecules in tight junction (TJ) complexes on cell membranes, while aquaporin-4 (AQP-4) is a kind of hydrophobic transmembrane protein in cell membrane to control water drainage in nervous system. The activity changes of these three proteins directly affect the structural integrity and the functional stability of BBB. In the present study, we aimed to investigate the effects of NBP on the ultrastructure changes of BBB and the expressions of AQP-4 and TJ- associated proteins, so as to reveal the underlying mechanisms of neuro-protection and the feasibility of NBP treatment in acute CO poisoning cases.

## Materials and Methods

### Experimental Model and Groups

A total of 144 adult healthy male Sprague-Dawley rats (230 ± 20 g) were obtained from Qingdao Academy of Medical Sciences, and implemented in accordance with the guidelines of Care and Use of Laboratory Animals issued by China National Institute of Health. All rats were quarantined in an animal room with a 12 h light/dark cycle for at least 7 days under standard conditions prior to experiment, and allowed access to food and water *ad libitum* intake. Afterward, rats were assigned into three groups as follows using a random number table: a normal control group (NC group, *n* = 48), a CO poisoning group (CO group, *n* = 48), and a NBP treatment group (NBP+CO group, *n* = 48). Rats both in CO group and NBP+CO group were exposed to 1000 ppm CO in air for 40 min and then to 3000 ppm for another 20 min in an animal oxygen chamber as described previously (Li Q. et al., 2015), while those in NC group were permitted to breathe fresh air. Carboxyhemoglobin (HbCO) concentration of arterial blood was detected after intraperitoneal injection 3% pentobarbital by a Blood Gas Analyzer (RapidLab, Bayer HealthCare, Germany). Rats with coma and high HbCO concentration (>40%) were considered as the successful models of acute CO poisoning. Core body temperature was maintained at 36∼37°C using a rectal probe during the whole experiment. The vital signs of rats were monitored every hour till consciousness returned to normal. Three rats were excluded in the final experimental statistics because of either the failure to recover consciousness (two rats) or a low concentration of HbCO (less than 40%, one rat). Meanwhile, another three rats met experimental requirements were enrolled in the relevant group. All experimental protocols and animal handling procedures were approved by the Animal Care and Use Committee of Affiliated Hospital of Qingdao University Medical College (Permit Number: 14-0027). All animals were treated with humane endpoints in the whole experiment process and euthanized by intraperitoneal injection of 3% pentobarbital to minimize animal suffering and distress when needed.

### Treatment Interventions

All rats were suffered hyperbaric oxygen therapy once a day in animal oxygen chamber after CO exposure until decapitated. The parameters set as follows: pure oxygen for 5 min, boost 20 min, 0.2 MPa oxygen 60 min, and finally decompression 20 min. The oxygen concentration was maintained between 95 and 99% in the animal cabin during hyperbaric oxygen therapy. NBP (Lot number: 11040311; purity: 100%; chemical formula: C_12_H_14_O_2_, molecular weight: 190.24) was kindly supported by Shijiazhuang Pharmaceutical, Co., Ltd, China. Rats in NBP+CO group were administrated orally NBP at a dose of 6 mg/100 g by gavage with a stomach tube at 2 h after CO exposure according to the previous studies (Zhao et al., 2013; Li J. et al., 2015), twice a day until sacrifice, and those in CO group and NC group were received the same dosage of pure olive oil as placebo at the same time.

### Determination of Brain Water Content

After interventions described above, brain water content was detected by dry–wet weight method (Chen et al., 2015). Briefly, four rats in each group were decapitated on days 1, 3, 7, and 14 after deeply anesthesia by 3% pentobarbital, respectively. Subsequently, the whole brain tissues were taken out from the skull and weighed first (wet weigh). Dry weight was obtained on an electronic balance after desiccation in an electric oven at 100°C for 24 h. Brain water content was then calculated as a percentage: brain water content (%) = [(wet weight – dry weight)/wet weight] × 100%.

### Transmission Electron Microscopy (TEM) with Lanthanum Tracer

Lanthanum tracer is often used for the study of cell connection and cell membrane permeability change in recent years (Kalachev, 2015; Yazama et al., 2015). In the present study, four rats in each group were intraperitoneally anesthetized at definite time points, and then were successively perfused 0.9% sodium chloride 200 ml for 1 h and 2% lanthanum nitrate-dimethyl arsenic 250 ml from left ventricular for 2 h. After stiffness of rat body, the whole brain was taken out, and cerebral cortex and hippocampus were isolated with craniotomy, respectively. Further, the tissues were cut into three sectors about 1 mm size and continuously fixed more than 2 h in a lanthanum nitrate fixative. After dehydration in ethanol and acetone and soak in 1% osmium tetroxide for 2 h, the brain sectors were embedded in E-pon 812 and thermally polymerized at 60°C for 48 h, finally cut into 90 nm specific ultrathin sections. With double staining of saturated uranyl acetate and citrate lead, the sections were put on copper nets (200 mesh), and were observed under a transmission electron microscope (TEM; JEM-100CX2, Japan). The BBB permeability and ultrastructural alterations were evaluated by the exudation of lanthanum nitrate.

### Immunohistochemical Assay

Sequential paraffin slices of brain tissues were prepared for immunohistochemical assay. ZO-1 (catalog number: sc-8146) monoclonal antibodies were purchased from Santa Cruz Company. All procedures were performed in accordance with the manufacturer’s protocols. ZO-1 antibody was diluted up to 1:200. The cells, with brown granular in membrane or cytoplasm under a light microscope (Leica, Germany), were considered as ZO-1 positive expression. Negative control slides were added 0.01 mmol/l phosphate buffered solution (PBS) instead of monoclonal antibody. Four non-overlapping fields were randomly observed in the left hemisphere, and the positive cells were calculated from four serial slices in each rat under a light microscope. Using a Leica Qwin image processing and analysis system, the optical density (OD) value of target protein in each view was determined and standardized by the negative control (Varghese et al., 2014; Tang et al., 2016).

### Immunofluorescence Staining

AQP-4 (catalog number: sc-9887) and claudin-5 (catalog number: sc-17668) monoclonal antibodies were both purchased from Santa Cruz Company. Four sequential paraffin sections of each rat were fixed in 4% paraformaldehyde at 4°C for 15 min, blocked non-specific binding sites for 2 h, and probed with primary monoclonal antibodies (anti-AQP-4 diluted 1:200 in PBS, anti- claudin-5 1:150) for 2 h at 37°C, fluorescent secondary antibodies for 1 h at 37°C, finally mounted with 50% glycerol. The whole process was carried out in a dark room and slides were washed fully with PBS as previously described (Lochhead et al., 2010). The positive cells were observed in four non-overlapping views under a fluorescence microscope (Leica, Germany), and the OD value in each view was determined by the Leica Qwin image processing and analysis system.

In order to determine the location relationship between ZO-1 and claudin-5, we used double immunofluorescence labeling in the present study. ZO-1 (SABC-FITC) was definited as the first colorating antibody, and claudin-5 (SABC-CY3) was as the second according to the shade of coloration. With the laser excitation and reception of different wavelength, ZO-1 (diluted 1:150) positive cells appeared yellow-green light, while claudin-5 (diluted 1:150) positive cells showed red light under a 400-fold fluorescence microscope. ZO-1 and claudin-5 positive cells were observed in the same view using different excitation wavelengths and merged image was obtained by photoshop7.0 software.

### Western Blot Analysis

Four rats in each group were deeply anesthetized and perfused at given time points as described above. The brain samples were separated and transferred to polyvinylidene fluoride membranes (Millipore, Billerica, MA, USA). After blocking with Tris-buffered saline and Tween 20 solution (TBST) containing 10% skimmed milk for 1 h, the membranes were incubated with primary antibody (ZO-1 dilution 1:550, claudin-5 dilution 1:500) for 30 min, and then were treated with horseradish peroxidase (HRP)-conjugated secondary antibody for 2 h at room temperature. Further, membranes were then washed with PBS and developed in X optical film according to the manufacturer’s instructions. The absorbance (A) value of target protein was assessed by a Bio-Rad 2000 gel imaging system and Quantity one software and normalized against β-actin value in the same specimen as an internal reference. All experiments were carried out in triplicate.

### Statistical Analysis

All experiments were repeated at least three times. Data were expressed as mean ± standard deviation (SD), and differences in the parameters were analyzed using one-way analysis of variance (ANOVA) followed by the least significant difference (LSD) *t*-test with SPSS 19.0 statistics software (IBM, Armonk, NY, USA). All tests were considered statistically significant at *P* < 0.05.

## Results

### Alterations in Brain Water Content

**FIGURE 2:**
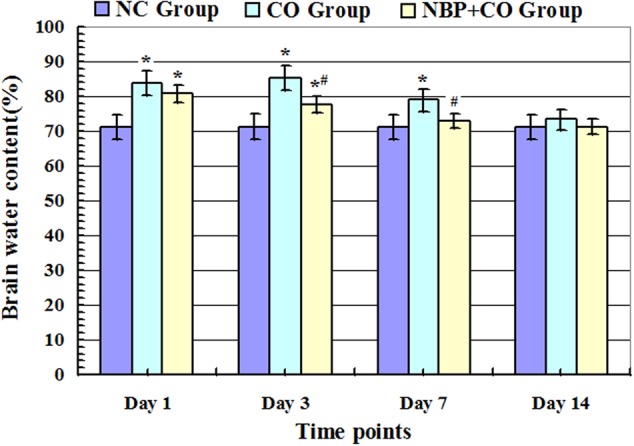
**Alterations of brain water content in NC, CO, and NBP+CO groups**. Brain water content in CO group was increased gradually on day 1 after exposure to CO, peaked on day 3 and then slightly decreased, yet it still exhibited a high level on day 7, and there were statistically differences in brain water content between day 1 and day 7 compared with that in normal control group (*n* = 4, *P* < 0.05). In the meantime, brain water content in NBP+CO group was also lower than that in CO group (*n* = 4, *P* < 0.05). ^∗^Compared with NC group, *P* < 0.05; ^#^compared with CO group at the same time point, *P* < 0.05 (*F* = 13.261∼28.782).

As shown in **Figure 2**, after acute exposure to CO, the brain water content of rats in CO group was increased gradually on day 1, peaked on day 3, and then gradually reduced. Compared with NC group, there were statistically differences at the same time points (*P* < 0.05). However, after NBP treatment, the brain water content obviously decreased, and there existed a significant difference versus CO poisoning rats on day 3 and day 7 (*P* < 0.05), respectively, suggesting that the early administration of NBP treatment can significantly attenuate brain edema, and to a large extent, efficiently prevent brain tissue against CO toxicity.

### Changes in BBB Permeability and Ultrastructure

**FIGURE 3:**
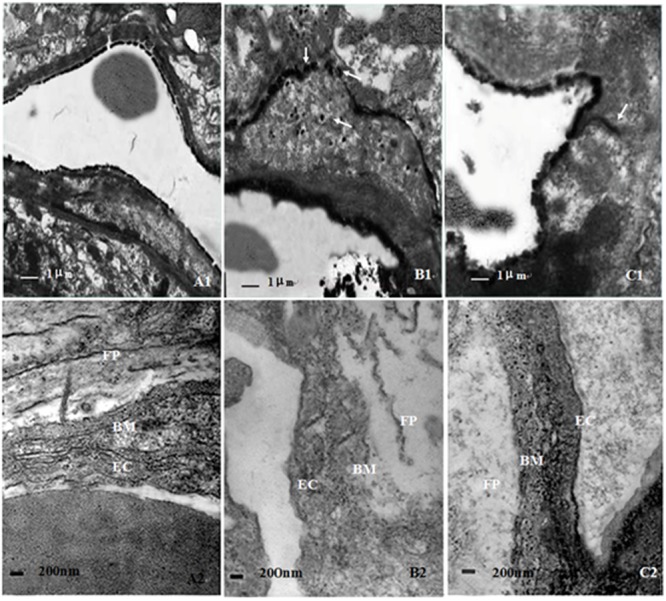
**Ultrastructure changes of blood–brain barrier in different groups.** The structure of BBB was relatively intact in NC group, and lanthanum nitrate particles were confined within the microvessel walls (A1,A2). However, the structural integrity of vascular walls was seriously destroyed in CO group, and lanthanum nitrate particles were penetrated from vessel walls into brain parenchyma (B1,B2). By contrast, the ultrastructural damage of microvessels was not serious in NBP treatment group, only a small part of lanthanum particles passed through the vessel wall and leaked into brain parenchyma (C1,C2). Arrows: lanthanum nitrate particles; Bm: basement membrane; Ec: endothelium cell; Fp: foot process; RBC: red blood cell (Scale bar is 1 μm in A1,B1,C1, and 200 nm in A2,B2,C2; *n* = 4).

The results of TEM showed that lanthanum nitrate particles were confined only within the microvessel walls of hippocampus and cortex in NC group, and little extravagated outside of microvessels. However, rats in CO group appeared significant cerebral edema and BBB dysfunction on day 1 and day 3 after CO poisoning. The structural integrity of vascular walls both in hippocampus and cortex was seriously destroyed, and lanthanum nitrate particles were apparently penetrated from vessel walls into brain parenchyma. Nevertheless, no lanthanum particles were observed in cytoplasm of endothelial cells in all electron microscopic views, indicating that lanthanum particles can leak through TJs between endothelial cells of capillaries to rat brain parenchyma after CO poisoning (**Figure 3**). In the meantime, the ultrastructural damage of microvessels in hippocampus and cortex were attenuated after NBP administration, and only a small part of lanthanum particles passed through the vessel wall and leaked into brain parenchyma, mainly located in TJs among vascular endothelial cells and even arrived to the basement membrane. These results suggest that NBP could attenuate cerebral edema, significantly improve BBB function and the ultrastructural integrity.

### Expression Levels of ZO-1

**FIGURE 4:**
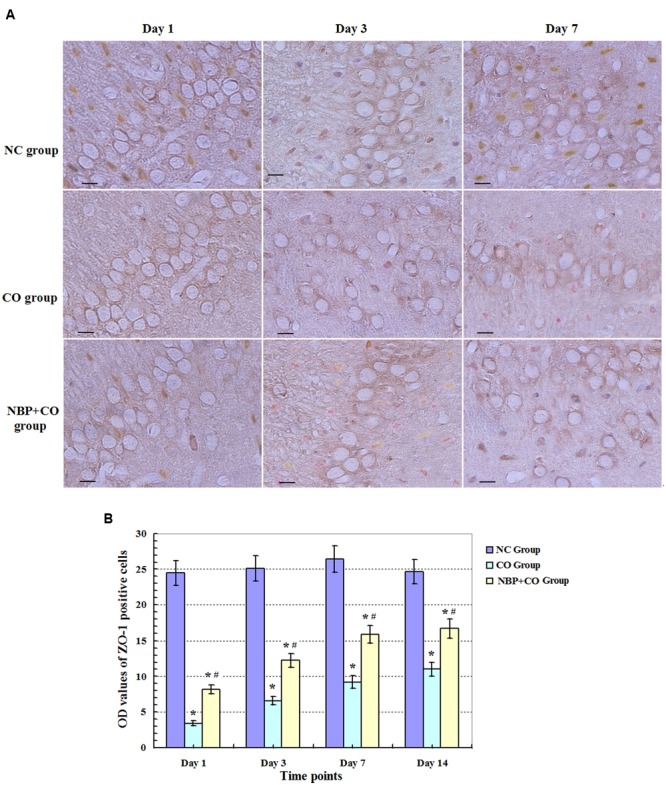
**Changes of ZO-1 protein in NC, CO, and NBP+CO groups.** ZO-1 protein-positive cells were observed in rat brain tissues in NC group, whereas the expression of ZO-1 protein was notably reduced on day 1, and still maintained at lower levels between day 3 and day 7 for CO poisoning subjects. However, the amount of ZO-1 positive cells was elevated in NBP treatment rats compared to that in CO group at the same time point (*n* = 4, *P* < 0.05). **(A)** The expressions of ZO-1 positive cells in each group; **(B)** The OD value alterations of ZO-1 positive cells among different groups (*n* = 4). Scale bar represents 30 μm. ^∗^ Compared with NC group, *P* < 0.05; **^#^** compared with CO group at the same time point, *P* < 0.05 (*F* = 15.516∼42.548).

**FIGURE 5:**
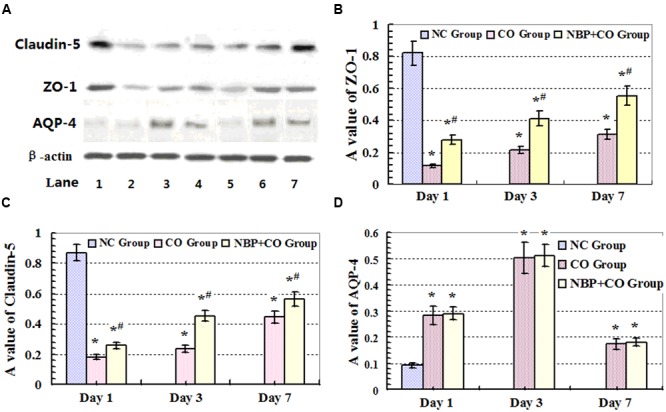
**Expressions of ZO-1, claudin-5 and AQP-4 proteins analyzed by Western blot.** High expression levels of ZO-1 and claudin-5 proteins were observed in rat brain tissue in NC group. CO poisoning can markedly decrease the expressions of ZO-1 and claudin-5 proteins both in CO group and NBP+CO group at early stage after exposure to CO. By contrast, NBP administration can significantly increase the levels of both ZO-1 and claudin-5 target proteins compared with those in CO group at the same time (*n* = 4, *P* < 0.05). **(A)** The expressions of ZO-1, claudin-5, AQP-4 and β-actin proteins by Western Blot assay. Lane 1: NC group; Lane 2: day 1 in CO group; Lane 3: day 3 in CO group; Lane 4: day 7 in CO group; Lane 5: day 1 in NBP+CO group; Lane 6: day 3 in NBP+CO group; Lane 7: day 7 in NBP+CO group. **(B)** The relative A values of ZO-1 protein; **(C)** The relative A values of claudin-5 protein; **(D)** The relative A values of AQP-4 protein. ^∗^Compared with NC group, *P* < 0.05; ^#^ compared with CO group at the same time point, *P* < 0.05 (*F* = 11.989∼33.472).

Under a light microscope with a 400-fold magnification, some ZO-1 positive cells with brownish yellow and different sizes and morphologies were observed in rat brain tissues in NC groups, and mainly located in the membrane and the cytoplasm. Meanwhile, we noted that the number of ZO-1 positive cells was markedly decreased on day 1 and maintained at relatively lower levels in hippocampus in CO poisoning rats between day 3 and day 14 in contrast to those in NC group (*P* < 0.05). Meanwhile, we also observed that the amount of ZO-1 positive cells was elevated in NBP treatment rats compared to those in CO group at the same time points (*P* < 0.05, **Figure 4**). Moreover, the same results were further confirmed using Western blot assay (**Figure 5**), indicating that NBP may play an important role in up-regulating the expression of ZO-1 protein against brain damage in acute CO-poisoning rats.

### Expression Levels of Claudin-5

**FIGURE 6:**
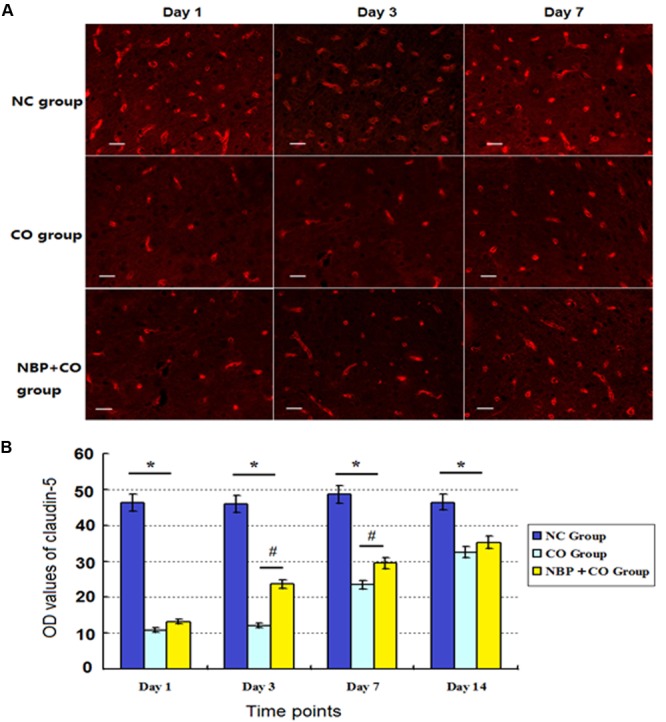
**Changes of claudin-5 expression in NC, CO and NBP+CO groups.** A large number of claudin-5-positive vessels were observed in NC subjects, and the positive levels of claudin-5 protein were notably reduced on day 1, followed by gradually restoration to approximate normal level on day 14 in CO group. The levels of claudin-5-positive vessel in NBP+CO group were higher than that in CO group (*n* = 4, *P* < 0.05). **(A)** The expressions of claudin-5 positive cells in each group; **(B)** The OD value alterations of claudin-5 positive cells in different groups (*n* = 4). ^∗^Compared with NC group, *P* < 0.05; ^#^compared with CO group at the same time point, *P* < 0.05 (*F* = 18.251∼32.817). Scale bar = 30 μm.

To some extent, the OD value of the marked positive vessels usually reflects the levels of BBB functional complex- associated proteins. In the present study, the intensity of immunofluorescent staining showed that a large number of claudin-5- positive vessels were observed in normal control subjects, and the positive levels of claudin-5 protein were notably reduced on day 1 after CO poisoning, followed by gradually restoring to approximate normal level on day 14 in CO group. Compared with the NC group, there were significantly statistical differences at definite times (*P* < 0.05). By contrast, the levels of claudin-5-positive vascular in NBP group were higher than that in CO group, and there existed statistically difference from day 3 to day 7 (*P* < 0.05, **Figure 6**). In addition, the same results were confirmed using Western blot analysis as shown in **Figure 5**.

### Changes of AQP-4 Expression in Rats after CO Poisoning

**FIGURE 7:**
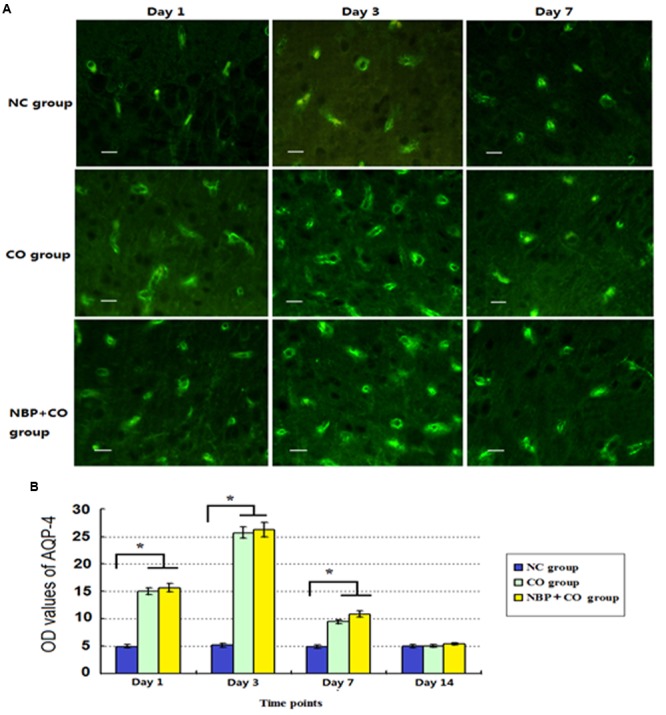
**The OD value alterations of AQP-4 expression in NC, CO, and NBP+CO groups.** A small amount of AQP-4 positive cells scattered in brain tissue in normal rats. After exposure to CO, AQP-4 expression was temporarily elevated and then gradually decreased. NBP treatment caused a slightly higher expression of AQP-4 protein compared to that in CO group, whereas it lacked a statistically significant difference between the two groups (*n* = 4, *P* > 0.05). **(A)** The expressions of AQP-4 positive cells in each group; **(B)** The OD value alterations of AQP-4 positive cells in different groups (*n* = 4). ^∗^Compared with NC group (*n* = 4, *P* < 0.05 (*F* = 10.621∼56.349). Scale bar = 30 μm.

Our results also revealed that a small amount of AQP-4 positive cells scattered in cerebral cortex, striatum and hippocampus in normal brain tissue. In CO group, AQP-4 positive cells increased on day 1, peaked on day 3, and then gradually decreased on day 7, finally remained at a lower level even on day 14 after CO poisoning (**Figure 7**). We noted that there were significant differences between CO group and NC group from day 1 to day 7 (*P* < 0.05). Furthermore, we also observed that the fluctuation of AQP-4 expression in NBP treatment subjects was consistent with that in CO group. Though AQP-4 levels were slightly higher than that in CO group at the same time points, it lacked a statistically significant difference between the two groups (*P* > 0.05).

### Relationships among ZO-1, Claudin-5 and AQP-4 Proteins

As mentioned above, claudin-5-positive cells in brain tissue were very rich and continuously expressed along cerebral vessels in NC group. Almost all of them were distributed in endothelial cells, especially in the outer lipid membrane of microvascular endothelial cells, suggesting that claudin-5 protein is closely related to the integrity of BBB. Unlike claudin-5, ZO-1 protein was located in cytoplasm, and under normal physiological conditions, ZO-1 protein was not only highly expressed in endothelial cells of BBB, but also in other cells of non-vessels barrier at a low level.

**FIGURE 8:**
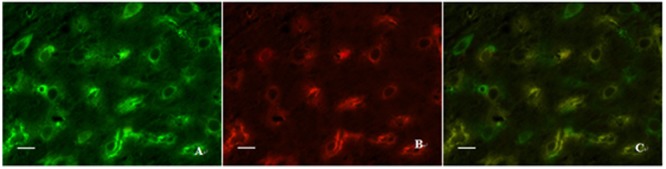
**Expressions of ZO-1 and claudin-5 in the same view using double immunofluorescence labeling. (A)** ZO-1 positive cells; **(B)** Claudin-5 positive cells; **(C)** Co-expression of ZO-1 and claudin-5 proteins (merged image). Scale bar is 30 μm.

Double immunofluorescence labeling was used to determine the location relationship between ZO-1 and claudin-5 in the present study. We found that almost all of claudin-5-positive cells also showed ZO-1 immunoreaction, but not all ZO-1 positive cells exerted claudin-5 immunogenicity in the same view (**Figure 8**). These results suggest that the two proteins cannot only co-exist in the same cells, but also express alone in different cells. Those ZO-1 positive cells which do not appear claudin-5 immunoreaction may be the microvascular endothelial cells of non-vessels barrier or other cell types.

**FIGURE 9:**
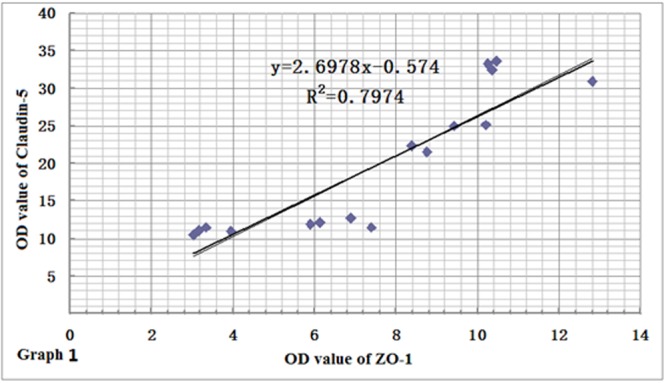
**Relationship between ZO-1 and claudin-5 protein expressions.** The variation of ZO-1 protein level was similar to that of claudin-5, and there existed positive correlation between the two proteins (*r* = 0.8930, *n* = 4).

**FIGURE 10:**
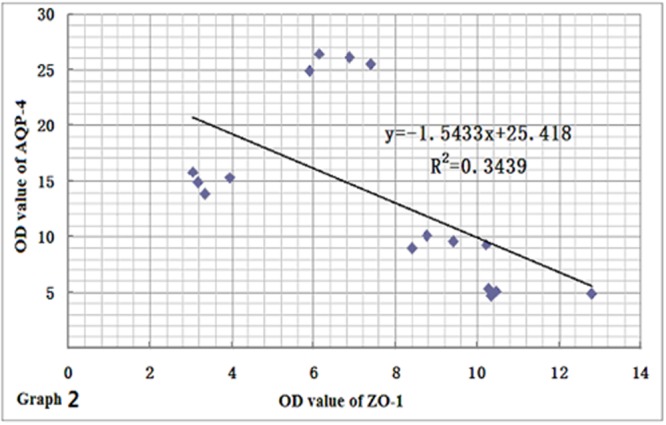
**Relationship between ZO-1 and AQP-4 protein expressions.** AQP-4 expression differed slightly from the fluctuation of ZO-1 protein, and there may exist negative correlation between the two proteins to some extent (*n* = 4, *r* = -0.5864).

In order to clarify the relationships among the expression levels of the three proteins, we performed a linear regression analysis. Based on the results of immunohistochemistry and immunofluorescence assay, we found that the expression levels of ZO-1 and claudin-5 proteins decreased rapidly in rats after CO poisoning. However, after rats got oxygen supply again, their expressions were consistently increased, and the level of ZO-1 was markedly correlated with that of claudin-5 by statistical analysis (*r* = 0.8930, **Figure 9**). This result was also consistent with that of Western blotting assay. In contrast to the expression variations of ZO-1 and claudin-5 proteins, AQP-4 expression was only temporarily elevated after CO poisoning in rats, further gradually dropped with oxygen supplement again. This result differed slightly in the fluctuation of ZO-1 protein and it may existed negative correlation to some extent (*r* = -0.5864, **Figure 10**).

## Discussion

**FIGURE 11:**
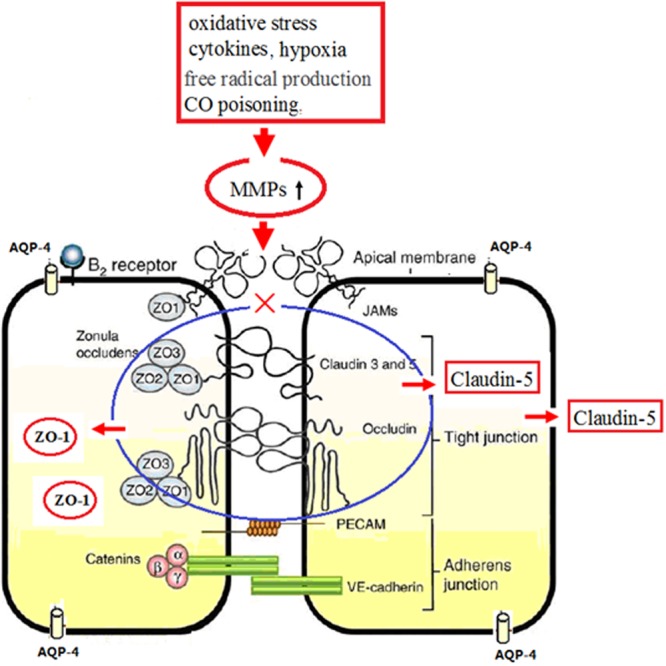
**Schematic diagram of ZO-1, claudin 5, and AQP-4 in continuous endothelium and the molecular mechanism after CO poisoning.** Physiologically, tight junction complexes (including ZO-1, occludin, and claudin-5 proteins) anchor in the transmembrane proteins of endothelial cells (blue mark). Under the pathological circumstances, such as oxidative stress, free radicals, CO poisoning, hypoxia and inflammation, neutrophils, and lymphocytes will induce the over-production and the activation of MMPs, which subsequently accelerate the lysis of TJ complexes and the degradation and translocation of ZO-1 and claudin-5 proteins, resulting in the impairment of BBB (red mark).

The BBB is a dynamic regulatory interface between capillaries and nerve tissue, whose role is to restrict the paracellular movement of ions and polar solutes from the blood to the brain, and contribute to the creation and maintenance of a specific brain homeostasis (Lehner et al., 2011). The barrier property of BBB primarily depends on the integrity of capillary endothelial cells [brain microvascular endothilial cells (BMECs)] and intercellular TJs (Ronaldson and Davis, 2012; Krueger et al., 2013). Tight connections are the basis of BBB located at the apical border of the lateral membrane as a permeability barrier and a fence that separate apical and baso-lateral domains within the plasma membrane of epithelial and endothelial cells. ZO-1 is a cytoplasmic attachment protein and locates at the cytoplasmic surface of endothelial cells in all vertebrates TJs. As a crucial unit of TJ complexes, ZO-1 interacts with occludin and claudin proteins anchoring the transmembrane proteins to cytoskeletal scaffold of endothelial cell, as well as actin protein (see **Figure 11**, Bauer et al., 2014). ZO-1 still serves as a recognition protein for TJ placement and a support structure for signal transduction (Dörfel and Huber, 2012). It has been demonstrated that subcellular localization of TJ in BMECs, for example, the functional and structural changes as well as the localization alteration of ZO-1 protein, would finally regulate the permeability of BBB. ZO-1 expression level is closely related to the opening-shutting status of BBB. The functional and structural changes of ZO-1 protein can lead to TJ dissociation, followed by the increased intercellular gaps and vascular permeability (Maiers et al., 2013). The down-regulation of ZO-1 expression often indicates the integrity damage of BBB.

Claudin-5 is a kind of specific protein in tight connection of brain endothelial cell, and has been proved to be the necessary condition for the formation of BBB, involving in the selective permeability of TJs and cell polarization, thus playing crucial roles in adjusting the permeability of BBB. In recent years, some scholars have reported the close relationship between claudin-5 and brain edema. Claudin-5 expression in brain tissue significantly increased after traumatic brain injury (Dong et al., 2013), and the down-regulation of claudin-5 expression often accompanied by the increased permeability of BBB in many experimental animal models of traumatic and ischemic brain injury and multiple sclerosis (Lanz et al., 2013). The expression of claudin-5 was reduced, followed by the increased expression of VEGF-A and the BBB breakdown, as well as the permeability changes of the blood–spinal cord barrier in autoimmune encephalomyelitis animal models. Despite these findings, Koto et al. (2007) insisted that the types of claudin directly determined the function status of BBB. Moreover, HIF-1α, a basic helix-loop-helix PAS transcription factor, acting as a master regulator of oxygen homeostasis by upregulating various genes, could also reduce the level of claudin-5 expression, and increase the permeability of low molecular weight compound next to cells (Koto et al., 2007).

In the present study, we noted that brain edema after CO exposure may be due to the decreased expressions of ZO-1 and claudin-5 proteins induced by CO poisoning. The elevated oxidative stress (free radical damage), the release of inflammatory cytokines, and the ischemia and hypoxia injury induced by CO poisoning, will induce the over-production and the activation of matrix metalloproteinases (MMPs), which subsequently accelerate the lysis of TJ complexes and the degradation of ZO-1 and claudin-5 proteins, further influence the levels and the locations of both ZO-1 and claudin-5, finally resulting in the impairment of BBB (see **Figure 11**). This result is consistent with the studies of Mark and Davis (2002), Lehner et al. (2011), Al Ahmad et al. (2012). Meanwhile, we also found that ZO-1 positive cells can express not only in BBB but also in non-vessels barrier, indicating that some ZO-1 protein does not directly involve in TJs of BBB. Thus, ZO-1 is not considered as an ideal specific TJ marker in BBB. However, ZO-1 protein is much sensitive to all stimulus signals that affect the BBB function, and the decrease of ZO-1 expression and activity will damage the structure integrity of TJ and the function stability of cells. Therefore, ZO-1 can be used as a reliable indicator of the functional status of TJs. The interactive effects of ZO-1 and claudin-5 proteins would influence the structural and functional integrity of BBB.

Aquaporin-4 is the main water channel protein, and often expressed in astrocytes and ependymal cells, particularly in glial membrane at the joints between the capillary and the pia mater, playing important roles in the structure and function stability of BBB and the removals of neurotransmitter. Physiologically, AQP-4 is the main channel providing water transport into the nervous system water compartments and across the BBB, while in many pathological experiments, it participates in the formation of secondary brain edema in animal models, such as brain trauma after cerebral ischemia/hemorrhage, inflammation, epilepsy, metabolic encephalopathy, and brain tumors (Papadopoulos et al., 2004; Wang et al., 2013). Nevertheless, it also caused lots of controversy about the experimental results and mechanisms. It was reported that AQP-4 gene silencing mice lack the susceptibility to cytotoxic brain edema caused by bacterial meningitis (Manley et al., 2000), and AQP-4 knockout could reduce the rate of water entering brain in cytotoxic edema models, while it would attenuate water transportation from brain in vasogenic edema. However, the overexpressed AQP-4 would ultimately aggravate cytotoxic brain swelling in transgenic mice, and inhibiting the expression or function of AQP-4 could suppress cytotoxic brain edema. It was reported that AQP-4 expression decreased at 24 h after hypoxia/ ischemia in coronary occlusion animals (Meng et al., 2004), while its levels rapidly declined in the middle cerebral artery occlusion, especially in the hypoxic zone that oxygen supply has not restored (Amiry-Moghaddam et al., 2003). Nevertheless, when cells exposed to oxygen again, the decreased AQP-4 expression could be reversed, and even significantly enhanced (Yamamoto et al., 2001). Newborn animal with a short choking of 3.5 min followed by oxygen supply 6 h appeared the lower AQP-4 expression in whole brain (Lee et al., 2008), whereas the systemic hypoxia for 2 h and then following oxygen supply for 9 h would increase AQP-4 expression in brainstem in adult rats (Kaur et al., 2006). Thus, the effects of hypoxia on AQP-4 expression *in vivo* test depend not only on the scope and extent of hypoxia, but also on the range and the time of oxygen supplement.

Our results showed that the dynamic change of AQP-4 protein expression after CO poisoning was consistent with the time of cerebral edema appearance, suggesting that AQP-4 plays various roles at different stages of the formation and dissipation in brain edema induced by CO poisoning. The increased expression of AQP-4 at an early stage of CO exposure (<3 days) might facilitate the formation and development of brain edema. After oxygen supplement again, the function of AQP-4 transportation gradually recovered, and the tolerance of nerve cells to ischemia and hypoxia increased, causing a stable homeostasis of intracellular environment and reduction of brain edema. That is to say, AQP-4 plays a two-way regulatory role in cerebral edema after CO poisoning (Li et al., 2008). As mentioned above, the structure of BBB is rather integrity and the transport of water molecules must depend on AQP-4 channel from blood into brain tissue in cytotoxic brain edema. As AQP-4 gene is knocked out or loses its dysfunction, water molecules cannot enter brain tissue, and brain edema is significantly reduced. Thus, AQP-4 can promote the formation and development of edema in cytotoxic brain damage. However, in vasogenic brain edema, the BBB integrity is seriously damaged, and water molecules leak directly from blood vessels into brain tissue space and lead to brain edema without the participation of AQP-4, and there was no direct relationship between the severity of brain edema and the expression level of AQP-4 (Tait et al., 2008), while the removal of water molecules from brain requires the mediation of AQP-4 channel, and this result may explain the differences of AQP-4 expression in many hypoxia models and CO poisoning experiments. In our study, the BBB integrity was significantly damaged after CO poisoning, and thus water molecules flow directly from BBB into brain tissue and accelerate the formation and development of brain edema. In other words, brain edema after CO poisoning may result from the common affection of cytotoxic and vasogenic factors.

The administration of NBP could significantly increase the expressions of ZO-1 and claudin-5 proteins, but did not affect the level of AQP-4 expression after CO poisoning, suggesting that NBP could attenuate brain edema via upregulating the expression of ZO-1 and claudin-5 proteins, maintaining the structural and functional integrity of TJs, and thus exerting the protective effect on BBB at the early stages after CO exposure. Nevertheless, whether NBP directly regulates the expression of AQP-4 or not needs to be further investigated.

## Conclusion

Carbon monoxide poisoning could damage the ultrastructure and function of the BBB of rats and thus leads to cerebral edema. ZO-1 and claudin-5 may be the important molecule targets in the structural and functional damage of BBB after CO poisoning. NBP could significantly attenuate cerebral edema induced by CO poisoning, markedly improve the integrity and function of BBB via upregulating the expressions of ZO-1 and claudin-5 proteins. Taken together, NBP may be a novel strategy for the treatment of acute CO poisoning clinically.

## Author Contributions

Conceived and designed the experiments: QL, YZ. Performed the experiments: QL, MZ, BL. Analyzed the data: DG. Contributed reagents/materials/analysis tools: WB. Wrote the paper: MB, DG.

## Conflict of Interest Statement

The authors declare that the research was conducted in the absence of any commercial or financial relationships that could be construed as a potential conflict of interest.
